# Peer review of "Endoplasmic Reticulum-Targeted Phototherapy Remodels the Tumor Immunopeptidome to Enhance Immunogenic Cell Death and Adaptive Anti-Tumor Immunity"

_pharmaceuticals, 2025, doi:10.3390/ph18040491_

Round 1
Reviewer 1 Report
Comments and Suggestions for Authors
This manuscript investigates the mechanism of ER-targeted phototherapy using the photosensitizer ER-Cy-pONO2, previously reported by the same group. The study is well-structured and presents sufficient results to support its conclusions. I recommend the manuscript for publication in its current form.
Author Response
Comments and Suggestions for Authors
This manuscript investigates the mechanism of ER-targeted phototherapy using the photosensitizer ER-Cy-poNO2, previously reported by the same group. The study is well-structured and presents sufficient results to support its conclusions. I recommend the manuscript for publication in its current form.
Reply: We are very grateful for your review in the whole process. Thank you very much for your consideration of this work.

Reviewer 2 Report
Comments and Suggestions for Authors
The authors have studied the impact of endoplasmic reticulum-targeted phototherapy on the antigenicity of dying tumor cells. They have demonstrated that the ER-targeted photosensitizer can significantly remodel the antigenic landscape of 4T-1 breast cancer cells. The following analysis revealed the upregulation of antigen presentation pathways, and increase of high-affinity MHC-I ligands. In vivo, the ER-targeted phototherapy elicited robust anti-tumor immunity and significantly inhibited tumor growth. Their finding established an enhanced anti-tumor immunity provided by ER-targeted phototherapy, providing a novel approach to suppress tumor progression. I believe this study is important to the field because it is under explored of ER-targeted phototherapy on tumor cells. The authors have performed professionally on both in vitro and in vivo work. I have made the comments below to help improve the quality of this manuscript.
- Figure 2 and Figure 3 showed robust T cell immune response and the activation of DCs. Have you analyzed the NK cells function in these mice? Because NK cells play an important role in the innate immune system, I am curious whether ER-targeted phototherapy could induce both innate and adaptive immune response.
- Figure 6d, what do you mean Blk in the first image of ELISPOT assay? Did you include a sample with 15 peptide mixed to stimulate T cells and measure IFN-r with ELISPOT assay?
- Have you done ELISPOT assay with tumor infiltrating T cells? So you will know the difference between control and treated mice regarding of immune response of tumor specific T cells.
Author Response
Comments and Suggestions for Authors
The authors have studied the impact of endoplasmic reticulum-targeted phototherapy on the antigenicity of dying tumor cells. They have demonstrated that the ER-targeted photosensitizer can significantly remodel the antigenic landscape of 4T-1 breast cancer cells. The following analysis revealed the upregulation of antigen presentation pathways, and increase of high-affinity MHC-I ligands. In vivo, the ER-targeted phototherapy elicited robust anti-tumor immunity and significantly inhibited tumor growth. Their finding established an enhanced anti-tumor immunity provided by ER-targeted phototherapy, providing a novel approach to suppress tumor progression. I believe this study is important to the field because it is under explored of ER-targeted phototherapy on tumor cells. The authors have performed professionally on both in vitro and in vivo work. I have made the comments below to help improve the quality of this manuscript.
- Figure 2 and Figure 3 showed robust T cell immune response and the activation of DCs. Have you analyzed the NK cells function in these mice? Because NK cells play an important role in the innate immune system, I am curious whether ER-targeted phototherapy could induce both innate and adaptive immune response.
Reply:Thank you for your encouraging comments and professional suggestions for our manuscript. Your feedback has provided valuable insights for future research directions. Phototherapy has been reported to induce both innate and adaptive immune responses [1]. The main objective of our study is to explore whether the ER-targeted phototherapy mediated by ER-Cy-poNO2 can alter the immunogenicity of 4T-1 cells to evoke an adaptive immune response in BALB/c mice, thereby achieving the benefits of a robust anti-tumor immune response. However, we did not analyze NK cell function in this study. We agree that NK cells play a critical role in the innate immune system, and their involvement in ER-targeted phototherapy is an important question. In future studies, we will investigate whether ER-targeted phototherapy activates innate immunity by assessing NK cell function, which will help to further elucidate the comprehensive immune response induced by this treatment.
- Figure 6d, what do you mean Blk in the first image of ELISPOT assay? Did you include a sample with 15 peptide mixed to stimulate T cells and measure IFN-r with ELISPOT assay?
Reply: Thank you for your comments and for pointing out the need to clarify the blk group. In Figure 6d, blk group refers to the blank control, which includes wells containing splenocytes and culture medium without any peptide stimulation. This control establishes baseline cytokine secretion levels and excludes non-specific background signals in the ELISPOT assay. We have revised the manuscript to explicitly define the blk/pos/neg group (lines 347-351).
Regarding the use of a 15-peptide mixture to stimulate T cells, we tested the immunogenicity of the 15-peptide mixture in vitro, as described in the methods section. However, as shown in Figure S1, the mixture induced excessive cytokine secretion, resulting in spot coalescence that exceeded the detection limit of the ELISPOT assay. This prevented accurate quantification of T cell responses, so we did not include a mixed group in subsequent in vivo experiments. The limitations section of the manuscript (lines 543-545) acknowledges the lack of exploration of synergistic effects among multiple immunopeptides. In future studies, we will optimize experimental conditions to investigate the contribution of mixed peptides to splenocyte stimulation.

Figure S1. ELISPOT images showed IFN-γ produced by the indicated 15 mixed peptides-primed BALB/c mice splenocytes restimulated with indicated peptide-pulsed BMDCs (BMDCs + 15 mixed peptides).
- Have you done ELISPOT assay with tumor infiltrating T cells? So you will know the difference between control and treated mice regarding of immune response of tumor specific T cells.
Reply: Thank you for your valuable comments and suggestion. ELISPOT analysis of tumor-infiltrating T cells could provide insights into T cell activation in the tumor microenvironment. However, this experiment was not performed in our study. Instead, we used flow cytometry to analyze the infiltration and activity of CD8⁺IFN-γ⁺ T cells and Treg cells in tumor tissues. In our current work, ELISPOT was used to assess the immunogenicity of 4T-1 cells and the activation of splenocytes stimulated by 4T-1 cells after ER-Cy-poNO2 treatment. We acknowledge the importance of analyzing tumor-infiltrating T cells and will consider incorporating this experiment in future studies to further clarify the immune response of tumor-specific T cells.
Reference:
1. Zhong, Y. T.;Qiu, Z. W.;Zhang, K. Y., et al. Plasma membrane targeted photodynamic nanoagonist to potentiate immune checkpoint blockade therapy by initiating tumor cell pyroptosis and depleting infiltrating b cells. Adv Mater. 2025, e2415078. http://doi.org/10.1002/adma.202415078. PMID: 40012447.

Reviewer 3 Report
Comments and Suggestions for Authors
I would like to express my appreciation for the in-depth research and insightful findings. I particularly found the integration of transcriptomic and immunopeptidomic analysis to identify high-affinity MHC-I ligands to be a novel and valuable approach. The study offers exciting possibilities for enhancing peptide-based cancer vaccines. However, I had a few questions that I hope you could clarify and few suggestions included to enhance the readers interest.
Comments:
- How does ER-Cy-poNO2 remodeling of the immunopeptidome contribute to the upregulation of MHC-I ligands?
- What are the advantages of using the DeepImmuTM Neoantigen Discovery platform for predicting immunogenicity of peptides?
- How does the IEDB analysis resource NetMHCpan-4.1 aid in determining MHC-I binding capacity of identified peptides?
- What parameters were optimized in the LC-MS/MS analysis for characterizing the immunopeptidome of ER-Cy-poNO2-treated tumor cells?
- How were transcriptome sequencing and KEGG enrichment analysis used to identify differentially expressed genes in ER-Cy-poNO2-treated cells?
- How does ER-targeted phototherapy influence the proportion of CD8+IFN-γ+ T lymphocytes in spleens and tumors?
- How does the binding affinity of upregulated peptides to different MHC-I alleles (H2-Kd, H2-Dd, H2-Ld) contribute to their immunogenic potential?
- What are the implications of increased IFN-γ secretion in ELISPOT assays following ER-Cy-poNO2-mediated phototherapy?
- What additional validation steps are necessary to confirm the antigen-specific activation of CD8+ T cells by IF4G3986-994 in vivo?
Suggestions:
- The article emphasizes peptide-based vaccines over tumor cell-based vaccines but lacks a detailed comparison of their clinical advantages and challenges.
- Lack of tetramer assays to detect IF4G3986-994-specific CD8+ T lymphocytes.
- Unexplored synergistic effects among multiple immunopeptides.
- The study links ER stress to immunogenic cell death (ICD) but lacks details on how ER-Cy-poNO2 modulates specific ICD-related signaling pathways.
Authors can expand on these points in the discussion.
Author Response
Comments and Suggestions for Authors
I would like to express my appreciation for the in-depth research and insightful findings. I particularly found the integration of transcriptomic and immunopeptidomic analysis to identify high-affinity MHC-I ligands to be a novel and valuable approach. The study offers exciting possibilities for enhancing peptide-based cancer vaccines. However, I had a few questions that I hope you could clarify and few suggestions included to enhance the readers interest.
Comments:
- How does ER-Cy-poNO2remodeling of the immunopeptidome contribute to the upregulation of MHC-I ligands?
Reply: Thank you very much for your valuable comments. Immunopeptidomics primarily focuses on the detection and analysis of peptides that bind to major histocompatibility complex (MHC) molecules, including the ligands of both MHC class I and MHC class II. MHC class I molecules primarily present endogenous antigenic peptides, which typically originate from the degradation products of intracellular proteins. These peptides are critical for immune surveillance, allowing the immune system to recognize and eliminate transformed cells [1]. In our study, immunopeptidomic analysis revealed that ER-targeted phototherapy mediated by ER-Cy-poNO2 significantly remodels the immunopeptidome of 4T1 cells. Specifically, 127 MHC-I ligands showed upregulated expression among the shared immunopeptides (Figure 5d). The relevant revisions have been added to lines 485-491 of the manuscript.
Futhermore, gene set enrichment analysis (GSEA) of transcriptome sequencing data demonstrated significant upregulation of the ANTIGEN PROCESSING AND PRESENTATION (MMU04612) pathway. Key genes involved in this pathway, such as Psme1, which contributes to the synthesis of MHC class I molecules, and Hspa1b, Hspa1a, Hsp90aa1, Hsp90ab1, which are involved in the processing and presentation of antigenic peptides, were significantly upregulated.
- What are the advantages of using the DeepImmuTM Neoantigen Discovery platform for predicting immunogenicity of peptides?
Reply: Thank you for your careful review. The DeepImmuTM Neoantigen Discovery platform offers several key advantages for predicting the immunogenicity of peptides, as demonstrated in recent studies [2].The reference has been added on line 667 of the manuscript. First, DeepImmuTM utilizes advanced deep learning algorithms to integrate multiple features critical for immunogenicity prediction, including peptide-MHC binding affinity, proteasomal cleavage, and antigen stability. This comprehensive approach significantly improves the accuracy of identifying T-cell-activating antigens compared to traditional methods. Second, the platform supports high-throughput screening, enabling the rapid identification of numerous potential neoantigens from large datasets of tumor-specific mutations. This greatly enhances the efficiency of neoantigen discovery, which is crucial for personalized immunotherapy. Third, DeepImmuTM aligns with the latest trends in immunopeptidomics research by integrating multi-omics data (e.g., genomics, transcriptomics, proteomics), which facilitates the identification of high-quality epitopes with greater precision [3]. Finally, its hybrid framework, combining deep learning and probabilistic modeling, along with experimental validation, ensures accurate, tumor-specific, and widely applicable neoantigen predictions. These features make DeepImmuTM a powerful tool for advancing personalized cancer immunotherapy.
- How does the IEDB analysis resource NetMHCpan-4.1 aid in determining MHC-I binding capacity of identified peptides?
Reply: NetMHCpan-4.1, an IEDB resource, is a powerful tool for determining the MHC-I binding capacity of peptides by leveraging advanced machine learning algorithms and integrating diverse datasets. It combines Binding Affinity (BA, e.g., IC50) and Mass Spectrometry Eluted Ligand (MS EL) data to enhance the accuracy of predictions. This dual-data approach not only models the theoretical binding potential of peptides to MHC-I molecules but also correlates these predictions with experimentally validated immune responses, thereby improving reliability [4]. For example, NetMHCpan-4.1 successfully identified high-affinity binders (IC50 < 500 nM) for SLA-1*0401 with a 93% success rate, demonstrating its robustness in predicting peptide-MHC interactions [5]. Additionally, its deep learning framework enables the modeling of complex binding patterns across diverse MHC alleles, making it a versatile tool for immunological research. In summary, NetMHCpan-4.1 aids in determining MHC-I binding capacity by integrating experimental and computational data, providing accurate and reliable predictions, and offering a user-friendly interface for deriving immunological insights from peptide sequences. The reference has been added on line 663 of the manuscript.
- What parameters were optimized in the LC-MS/MS analysis for characterizing the immunopeptidome of ER-Cy-poNO2-treated tumor cells?
Reply: Thank you for your comments, and we have revised the manuscript accordingly (lines 616-624). In the LC-MS/MS analysis for characterizing the immunopeptidome of ER-Cy-poNO2-treated tumor cells, several parameters were optimized to enhance data quality and depth of analysis. Specifically, the mass spectrometer operated in data-dependent acquisition mode with a full mass scan range of m/z 100-1700, 10 MS/MS scans per cycle, a total cycle time of 2.27 seconds, and an ion intensity threshold of 2500 for triggering MS/MS. These optimizations were designed to maximize the detection of low-abundance peptides, improve the signal-to-noise ratio, and ensure the acquisition of high-quality spectra. As a result, these adjustments enabled a more comprehensive and precise characterization of ER-Cy-poNO2-induced immunopeptidome changes, particularly in the context of MHC-I upregulation and antigen presentation.
- How were transcriptome sequencing and KEGG enrichment analysis used to identify differentially expressed genes in ER-Cy-poNO2-treated cells?
Reply: Thank you for your comments. Thank you for your comments. To investigate the molecular mechanisms by which ER-Cy-poNO2- mediated phototherapy induces immunogenic cell death (ICD), transcriptome sequencing was performed to identify differentially expressed genes (DEGs), and KEGG enrichment analysis was used to uncover the biological pathways significantly associated with these DEGs.In transcriptome sequencing analysis, total RNA was isolated from both the ER-Cy-poNO2 + Laser treated and control 4T-1 cells. Libraries were constructed and sequenced on the Illumina platform with paired-end sequencing, using “Mus_musculus.GRCm39.dna.primary_assembly.fa” as the reference genome (Table S1). Reads were mapped to the mouse reference genome using STAR, and gene expression levels were quantified by FPKM. DESeq2 was then used to detect DEGs with significant expression changes (Log2 FoldChange > 1 and adjusted P-value < 0.05).
For KEGG pathway enrichment, DEGs were annotated using the KEGG database, and significantly enriched pathways were identified based on FDR < 0.05. Key pathways related to MHC-I antigen presentation, TNF signaling, and oxidative stress response were highlighted to explore ER-Cy-poNO2’s role in immune regulation and MHC-I ligand upregulation (Figure 4). This integrated approach revealed that ER-Cy-poNO2 activates transcriptional programs affecting MHC-I ligand expression, proteasome activity, and cross-presentation pathways, linking transcriptomic changes to immunopeptidome remodeling. The reference has been added on line 613 of the manuscript.

Table S1. Reference genomic information.
- How does ER-targeted phototherapy influence the proportion of CD8+IFN-γ+T lymphocytes in spleens and tumors?
Reply: Thank you for your comments. ER-targeted phototherapy increases the proportion of CD8⁺IFN-γ⁺ T lymphocytes in spleens and tumors by inducing immunogenic cell death (ICD) and enhancing antigen presentation. In tumor cells, ER-targeted phototherapy generates ROS, triggering ER stress and ICD, which releases tumor-associated antigens (TAAs) and damage-associated molecular patterns (DAMPs). TAAs are processed and presented by dendritic cells (DCs) to CD8⁺ T cells, activating them to secrete IFN-γ. Simultaneously, DAMPs activate pattern recognition receptors (PRRs) on DCs [6], further promoting CD8⁺IFN-γ⁺ T cell activation[7]. In the spleen, tumor antigens carried via the bloodstream are captured by antigen-presenting cells (APCs) such as DCs. These APCs process the antigens, load them onto MHC molecules, and present them to T cells. Upon recognizing the MHC-antigen complex, CD8⁺ T cells are activated into cytotoxic CD8⁺IFN-γ⁺ T cells. In the tumor microenvironment, ER-targeted phototherapy kills tumor cells, releasing TAAs and increasing antigen concentration. This promotes the recruitment of CD8⁺IFN-γ⁺ T cells from the spleen to the tumor, where they recognize MHC-I-presented antigens and exert cytotoxic effects [8]. Collectively, these processes enhance the activation and migration of CD8⁺IFN-γ⁺ T cells, increasing their proportion in both spleens and tumors and strengthening the anti-tumor immune response.
- How does the binding affinity of upregulated peptides to different MHC-I alleles (H2-Kd, H2-Dd, H2-Ld) contribute to their immunogenic potential?
Reply: Thank you for your comments. The binding affinity of upregulated peptides to MHC-I alleles like H2-Kd, H2-Dd and H2-Ld plays a critical role in determining their immunogenic potential. High-affinity peptides form stable peptide-MHC-I (pMHC-I) complexes, which are essential for efficient recognition by cytotoxic T lymphocytes (CTLs) and subsequent activation of CD8⁺ T cells. Each MHC-I allele has distinct binding motifs that influence peptide selection. For example, H2-Kd favors hydrophobic or basic residues at P2, while H2-Dd prefers acidic residues at P1. These allele-specific preferences shape the repertoire of presented peptides and the diversity of the T-cell response [9]. Stable pMHC-I complexes resulting from high-affinity binding enhance CD8⁺ T cell activation and IFN-γ production, driving a robust anti-tumor immune response. [10]. The relevant revisions have been added to lines 498-501 of the manuscript.
- What are the implications of increased IFN-γsecretion in ELISPOT assays following ER-Cy-poNO2-mediated phototherapy?
Reply: Thank you for your comments. IFN-γ is a key cytokine secreted by activated T cells and natural killer (NK) cells, playing a central role in anti-tumor immunity. Increased IFN-γ secretion in ELISPOT assays reflects a robust cellular immune response, indicating enhanced activation of immune cells such as CD8⁺ T cells. These cells recognize tumor antigens and secrete IFN-γ to promote tumor cell killing and inhibit tumor growth and metastasis [11]. Consequently, the increase in IFN-γ secretion detected by ELISPOT assay is of great significance following ER-Cy-poNO2 -mediated phototherapy. ER-Cy-poNO2-mediated phototherapy leads to the immunogenic remodeling of 4T-1 cells. Through the induction of endoplasmic reticulum (ER) stress and immunogenic cell death (ICD), 4T-1 cells are transformed from an immune evasion state to an immunodominant state, thereby enhancing antigen processing and MHC-I peptide presentation, and effectively cross-presenting to immune cells. This leads to significant activation of CD8⁺ T cells against antigens derived from phototherapy-modified tumor cells, as reflected by increased IFN-γ secretion and enhanced cytotoxicity. Additionally, local IFN-γ secretion regulates the tumor microenvironment by recruiting effector T cells and suppressing immunosuppressive factors such as TGF-β and IL-10 [12]. This reverses immune tolerance within the tumor microenvironment, further amplifying the anti-tumor immune response.
9.What additional validation steps are necessary to confirm the antigen-specific activation of CD8+ T cells by IF4G3986-994 in vivo?
Reply: Thank you for your comments. To further confirm the antigen-specific activation of CD8+ T cells by IF4G3986-994 in vivo, the following additional validation steps could be carried out:
- Tetramer assay: Perform a tetramer assay to detect IF4G3986-994-specific CD8⁺ T lymphocytes in BALB/c mice. This assay will directly identify antigen-specific T cells and clarify their presence and frequency in vivo.
- Quantification of cytotoxic molecules: Measure the expression levels of cytotoxic molecules such as perforin and granzyme in CD8⁺ T cells following stimulation with IF4G3986-994. This will provide functional evidence of the cytotoxic activity of antigen-specific T cells.
- Signaling pathway analysis:Some experiments related to signaling pathways should be carried out to further validate the mechanism of action of IF4G3986-994. These experiments address the current limitations of our study.
We will include these points in the limitations section of the manuscript (lines 540-543) and plan to conduct these experiments in future studies to strengthen the scientific rigor and continuity of our work.
Suggestions:
The article emphasizes peptide-based vaccines over tumor cell-based vaccines but lacks a detailed comparison of their clinical advantages and challenges.
Lack of tetramer assays to detect IF4G3986-994-specific CD8+ T lymphocytes.
Unexplored synergistic effects among multiple immunopeptides.
The study links ER stress to immunogenic cell death (ICD) but lacks details on how ER-Cy-poNO2 modulates specific ICD-related signaling pathways.
Authors can expand on these points in the discussion.
Reply:Thank you for your comments and providing good suggestions for our manuscript. We have revised our manuscript in aspect of discussion according to your suggestion. We have incorporated these suggestions into the discussion section of the manuscript (lines 537-550). We will continue to investigate these issues in future research and strive to overcome the above - mentioned limitations.
1. Sirois, I.;Isabelle, M.;Duquette, J. D., et al. Immunopeptidomics: Isolation of mouse and human mhc class i- and ii-associated peptides for mass spectrometry analysis. J Vis Exp. 2021. http://doi.org/10.3791/63052. PMID: 34723952.
2. Li, G.;Mahajan, S.;Ma, S., et al. Splicing neoantigen discovery with snaf reveals shared targets for cancer immunotherapy. Sci Transl Med. 2024, 16, eade2886. http://doi.org/10.1126/scitranslmed.ade2886. PMID: 38232136.
3. Li, G.;Iyer, B.;Prasath, V. B. S., et al. Deepimmuno: Deep learning-empowered prediction and generation of immunogenic peptides for t-cell immunity. Brief Bioinform. 2021, 22. http://doi.org/10.1093/bib/bbab160. PMID: 34009266.
4. Bonsack, M.;Hoppe, S.;Winter, J., et al. Performance evaluation of mhc class-i binding prediction tools based on an experimentally validated mhc-peptide binding data set. Cancer Immunol Res. 2019, 7, 719-736. http://doi.org/10.1158/2326-6066.Cir-18-0584. PMID: 30902818.
5. Pedersen, L. E.;Harndahl, M.;Rasmussen, M., et al. Porcine major histocompatibility complex (mhc) class i molecules and analysis of their peptide-binding specificities. Immunogenetics. 2011, 63, 821-34. http://doi.org/10.1007/s00251-011-0555-3. PMID: 21739336.
6. Carroll, S. L.;Pasare, C.;Barton, G. M. Control of adaptive immunity by pattern recognition receptors. Immunity. 2024, 57, 632-648. http://doi.org/10.1016/j.immuni.2024.03.014. PMID: 38599163.
7. Mellman, I.;Chen, D. S.;Powles, T., et al. The cancer-immunity cycle: Indication, genotype, and immunotype. Immunity. 2023, 56, 2188-2205. http://doi.org/10.1016/j.immuni.2023.09.011. PMID: 37820582.
8. Yang, K.;Halima, A.;Chan, T. A. Antigen presentation in cancer - mechanisms and clinical implications for immunotherapy. Nat Rev Clin Oncol. 2023, 20, 604-623. http://doi.org/10.1038/s41571-023-00789-4. PMID: 37328642.
9. Chiaro, J.;Antignani, G.;Feola, S., et al. Development of mesothelioma-specific oncolytic immunotherapy enabled by immunopeptidomics of murine and human mesothelioma tumors. Nat Commun. 2023, 14, 7056. http://doi.org/10.1038/s41467-023-42668-7. PMID: 37923723.
10. Rasmussen, M.;Fenoy, E.;Harndahl, M., et al. Pan-specific prediction of peptide-mhc class i complex stability, a correlate of t cell immunogenicity. J Immunol. 2016, 197, 1517-24. http://doi.org/10.4049/jimmunol.1600582. PMID: 27402703.
11. Yang, P.;Chen, Z.;Zhang, J., et al. Evaluation of varicella-zoster virus-specific cell-mediated immunity by interferon-γ enzyme-linked immunosorbent assay in adults ≥50 years of age administered a herpes zoster vaccine. J Med Virol. 2019, 91, 829-835. http://doi.org/10.1002/jmv.25391. PMID: 30613990.
12. Yamashita, N.;Long, M.;Fushimi, A., et al. Muc1-c integrates activation of the ifn-γ pathway with suppression of the tumor immune microenvironment in triple-negative breast cancer. J Immunother Cancer. 2021, 9. http://doi.org/10.1136/jitc-2020-002115. PMID: 33495298.
