# Supplementary material for: Endoplasmic Reticulum-Targeted Phototherapy Remodels the Tumor Immunopeptidome to Enhance Immunogenic Cell Death and Adaptive Anti-Tumor Immunity
Source: Pharmaceuticals (Basel). 2025 Mar 28;18(4):491. doi: 10.3390/ph18040491 (PMC12030737; doi:10.3390/ph18040491)
Supplement: Supplementary file 1 [file pharmaceuticals-18-00491-s001.zip › Supplementary Information.docx]

**1. Supplementary Figures**

**
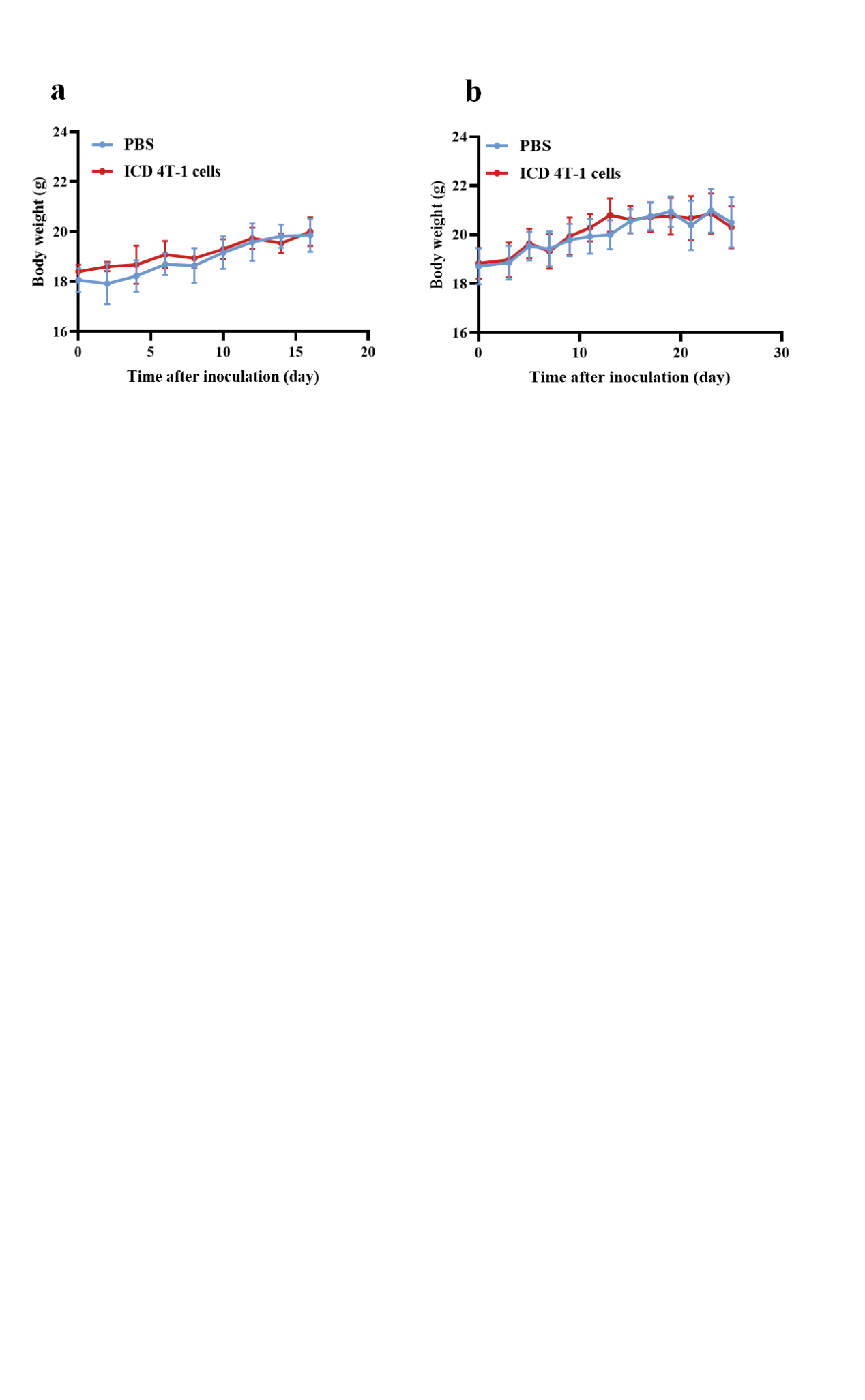
**

**Figure S1.** (**a)** Body weights of mice during the observation period in tumor vaccine experiments (n =5, mean ± s.d.). (**b)** Body weights of mice during the observation period in tumor treatment experiments (n =5, mean ± s.d.).


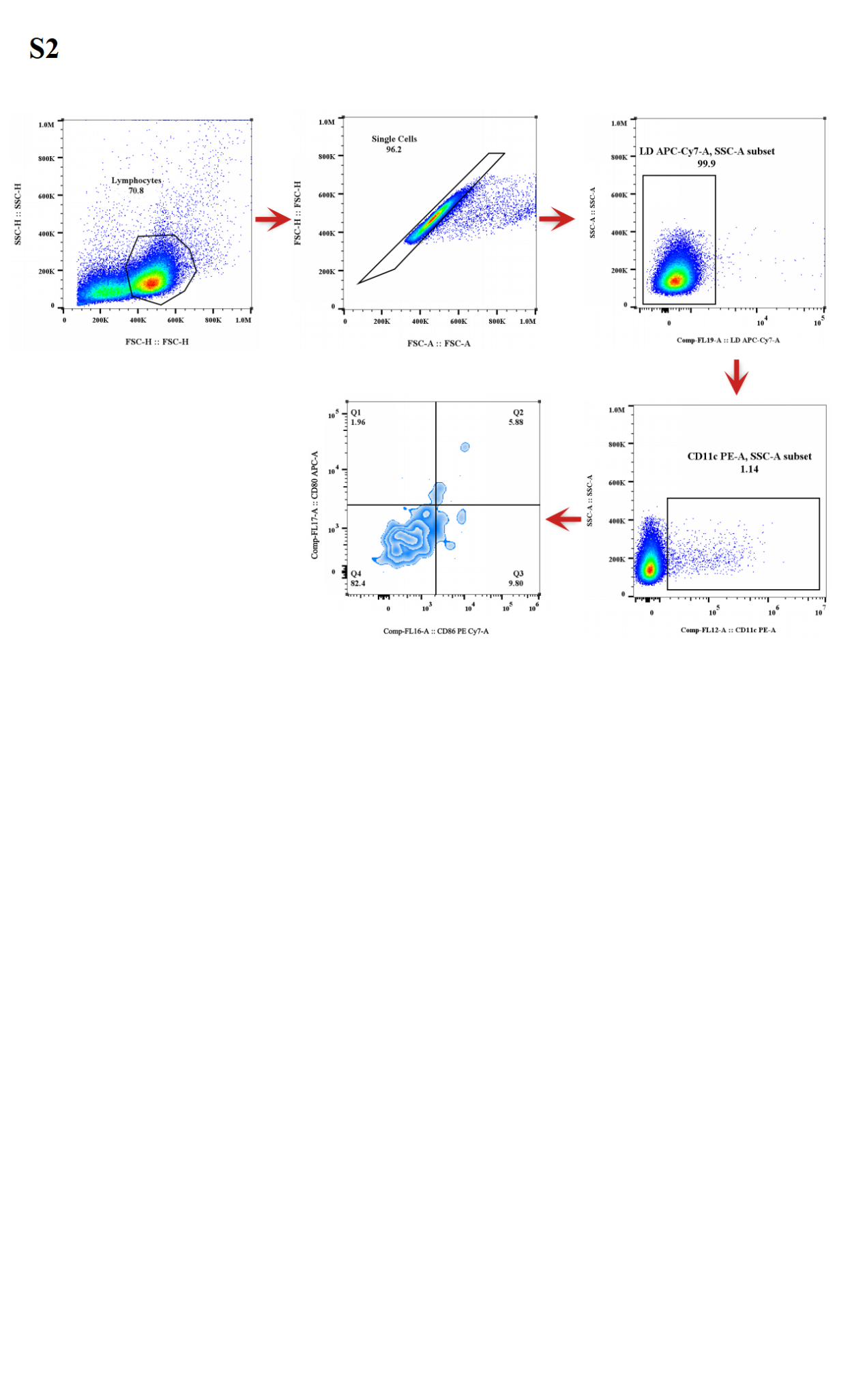


**Figure S2.** Gating strategy for detecting the proportion of mature DCs (CD80^+^CD86^+^) in Lymph Nodes.


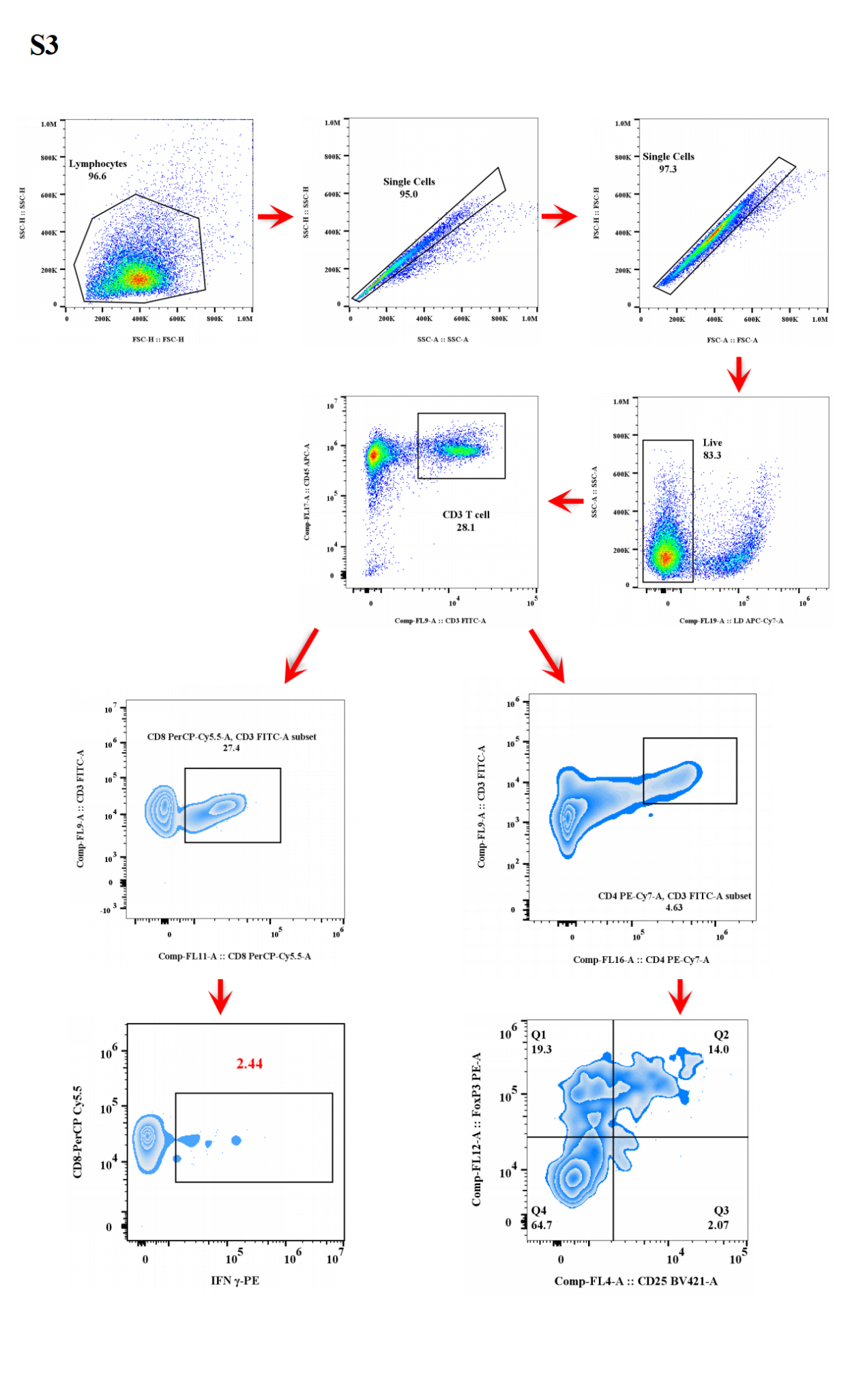


**Figure S3.** Gating strategy for detecting the proportion of CD3^+^CD8^+^ T lymphocytes, CD8^+^IFN-γ^+^ T lymphocytes in spleen and CD3^+^CD4^+^ T lymphocytes, CD25^+^Foxp3^+^ regulatory T cells in tumor.


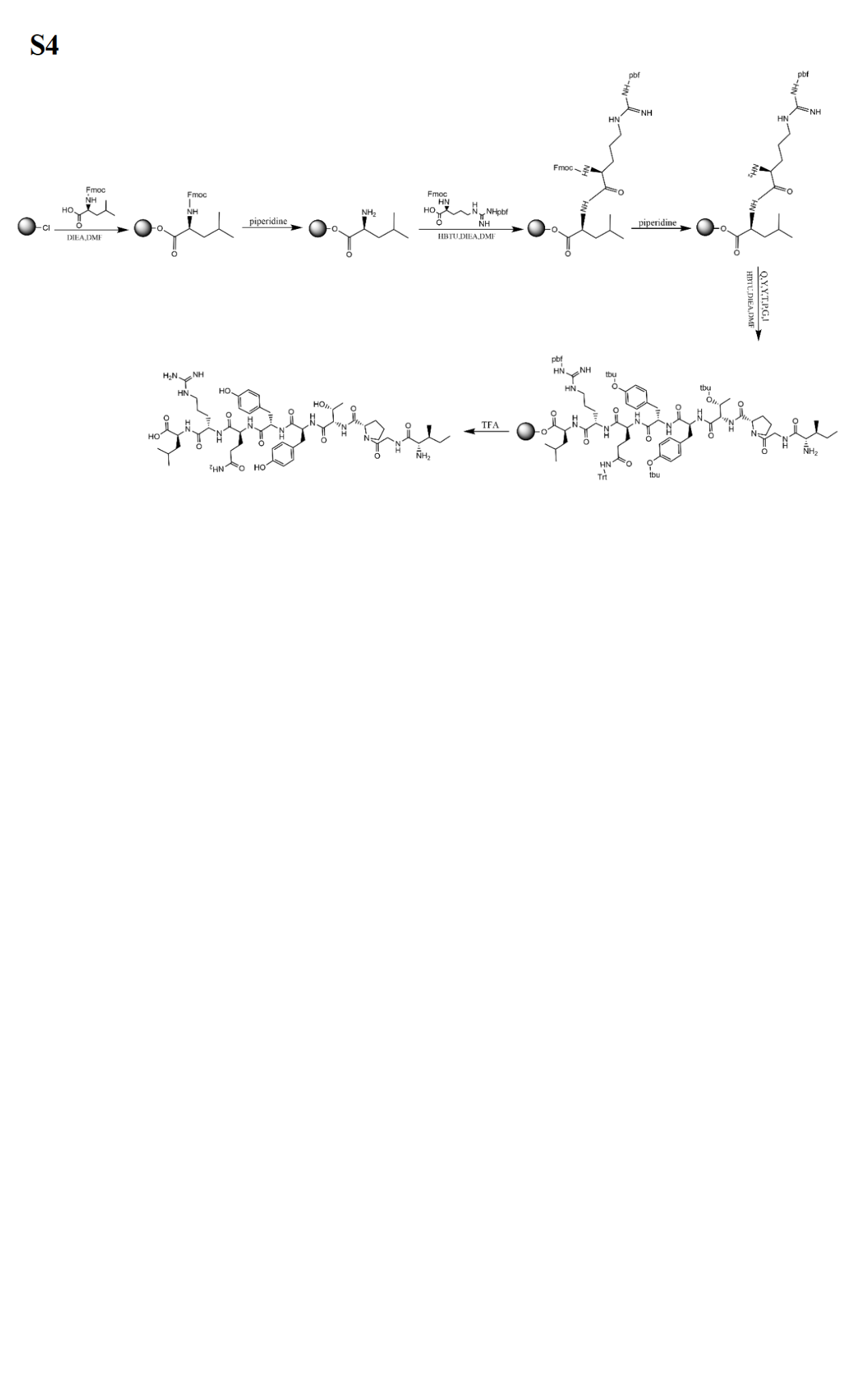


**Figure S4.** Flowchart of candidate peptide synthesis, Taking Polr2b_1043-1051_ (IGPTYYQRL) as an Example.


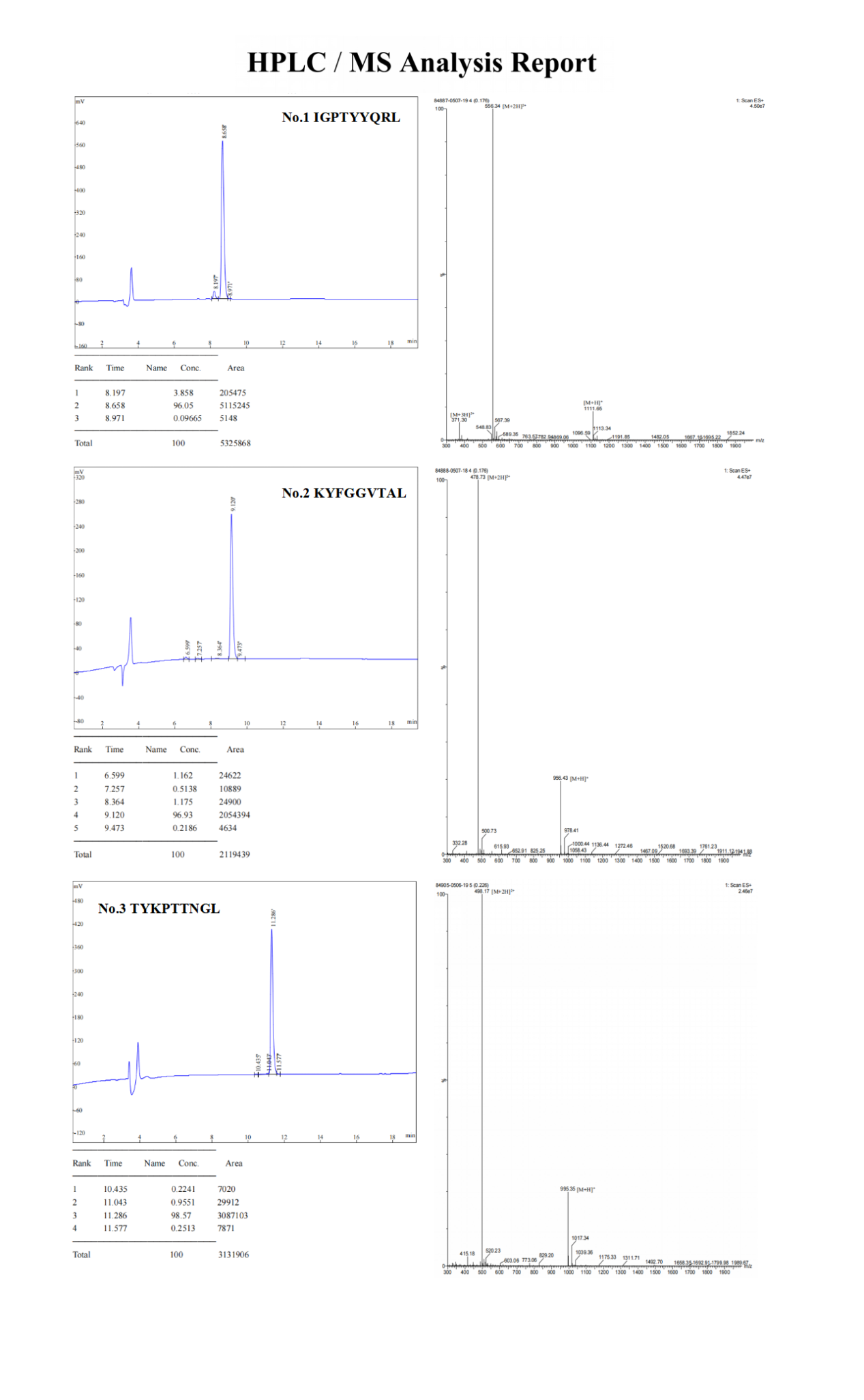
**Figure S5.** The mass spectrum (MS) and high-performance liquid chromatography (HPLC) chromatogram of No.1-3 candidate peptides.


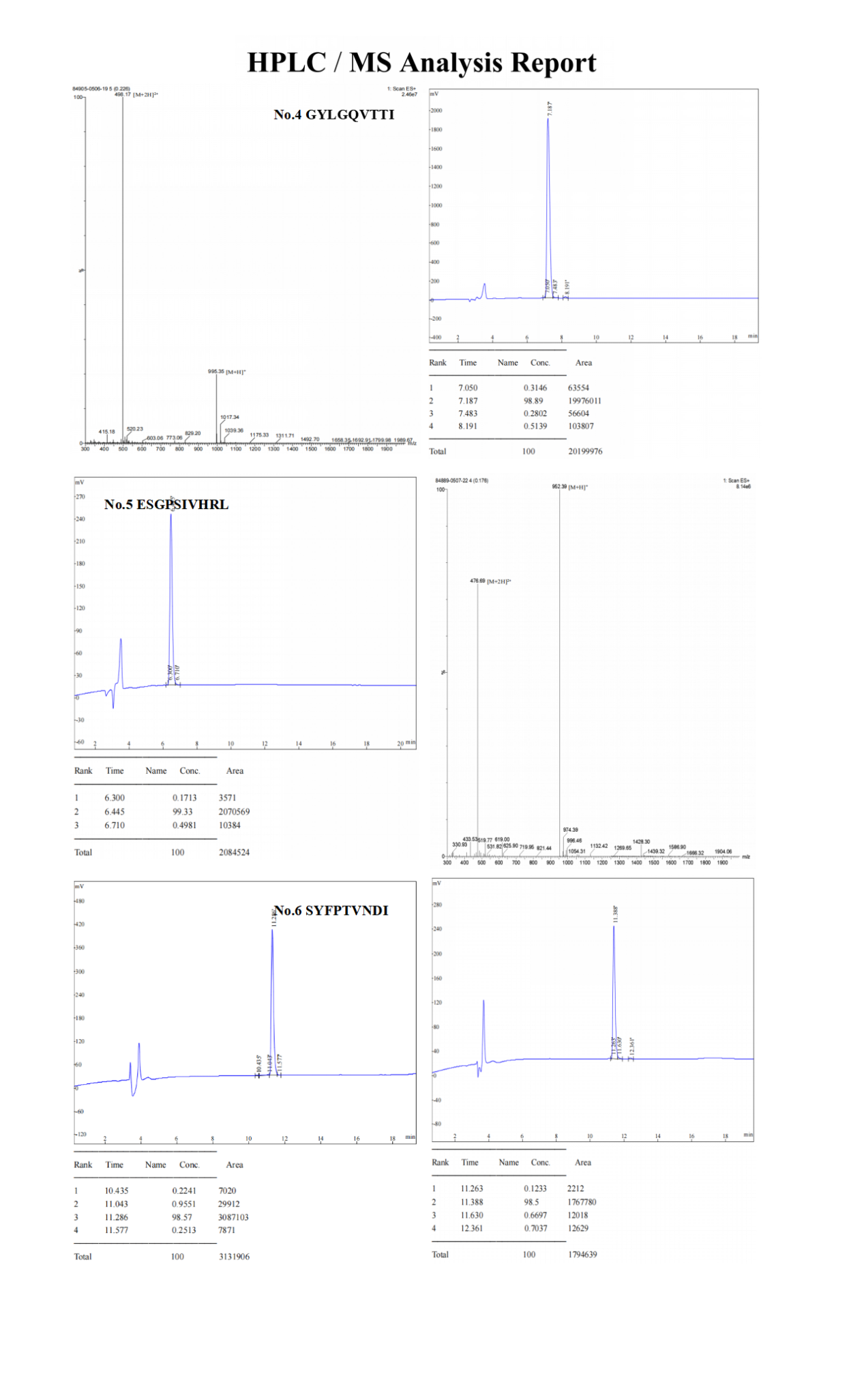
**Figure S6.** The MS and HPLC chromatogram of No.4-6 candidate peptides.


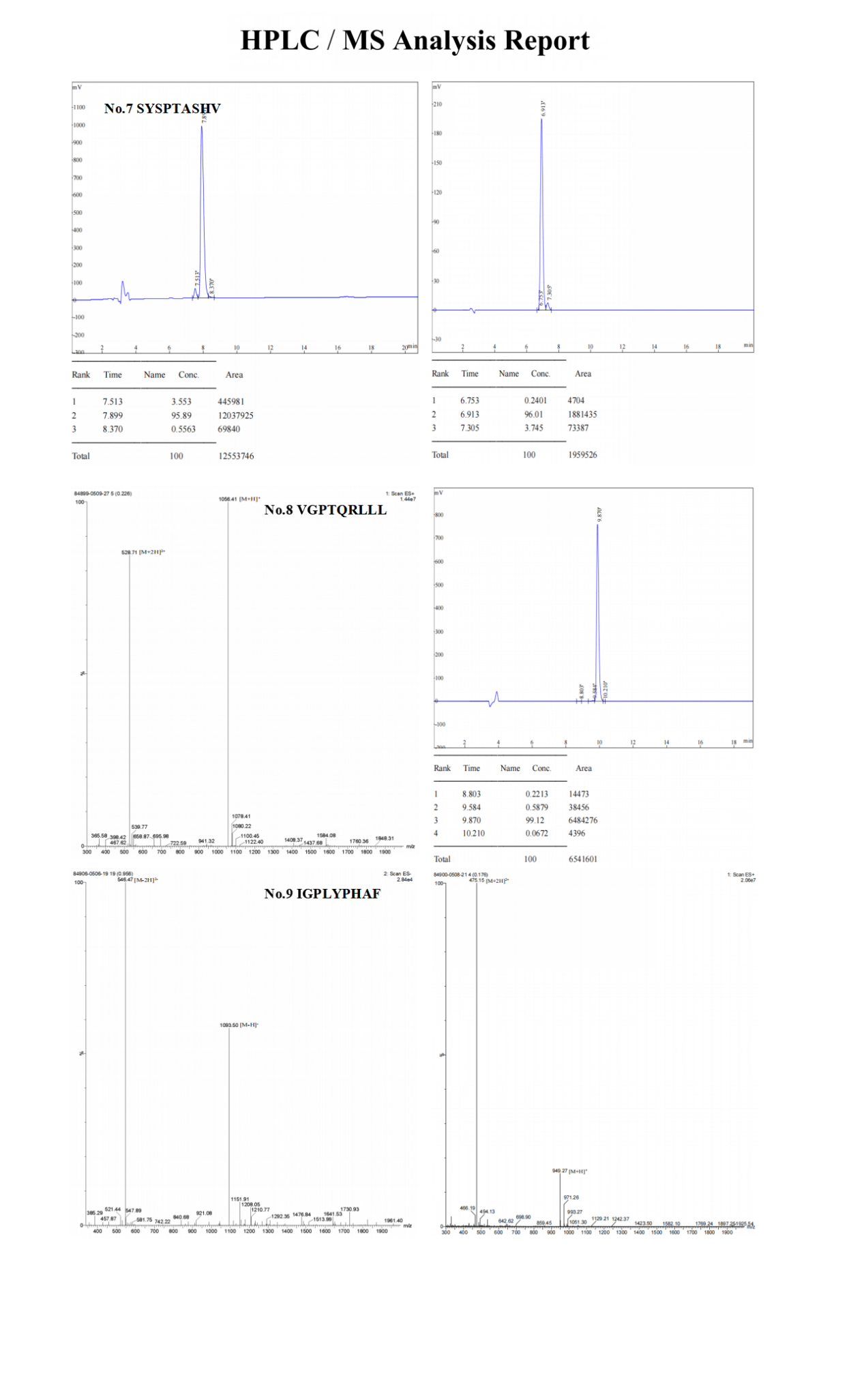
**Figure S7.** The MS and HPLC chromatogram of No.7-9 candidate peptides.


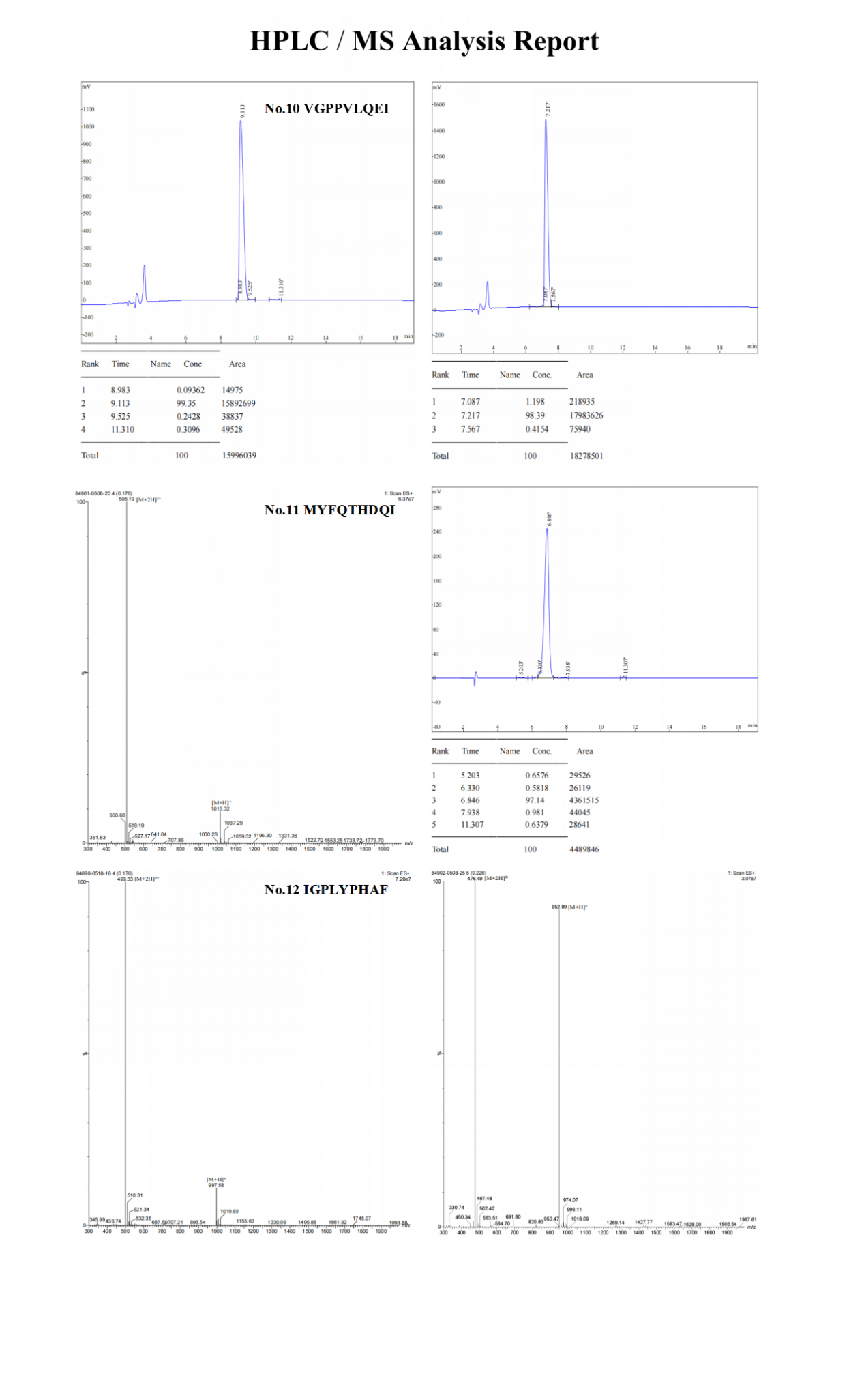


**Figure S8.** The MS and HPLC chromatogram of No.10-12 candidate peptides.


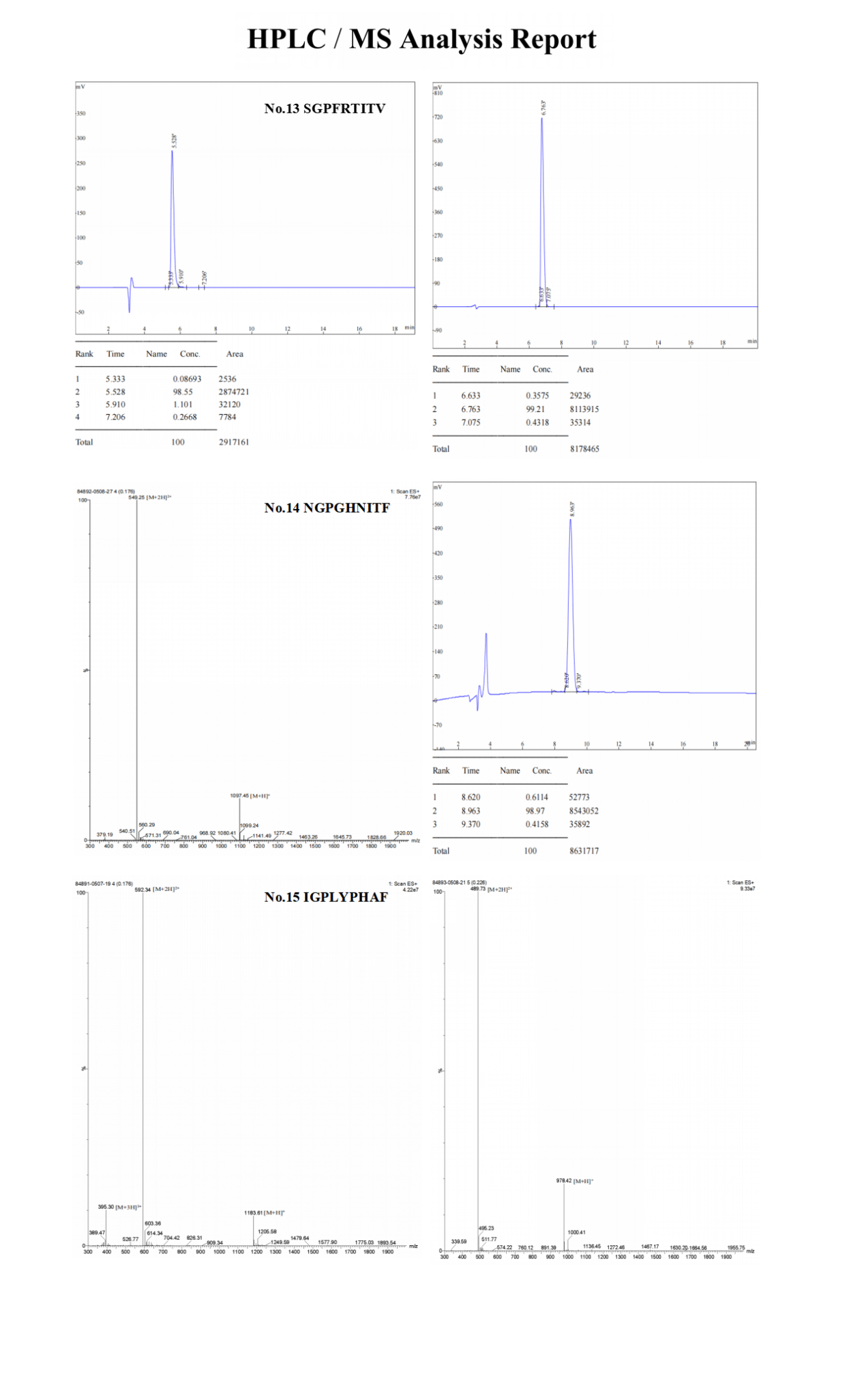


**Figure S9.** The MS and HPLC chromatogram of No.13-15 candidate peptides.


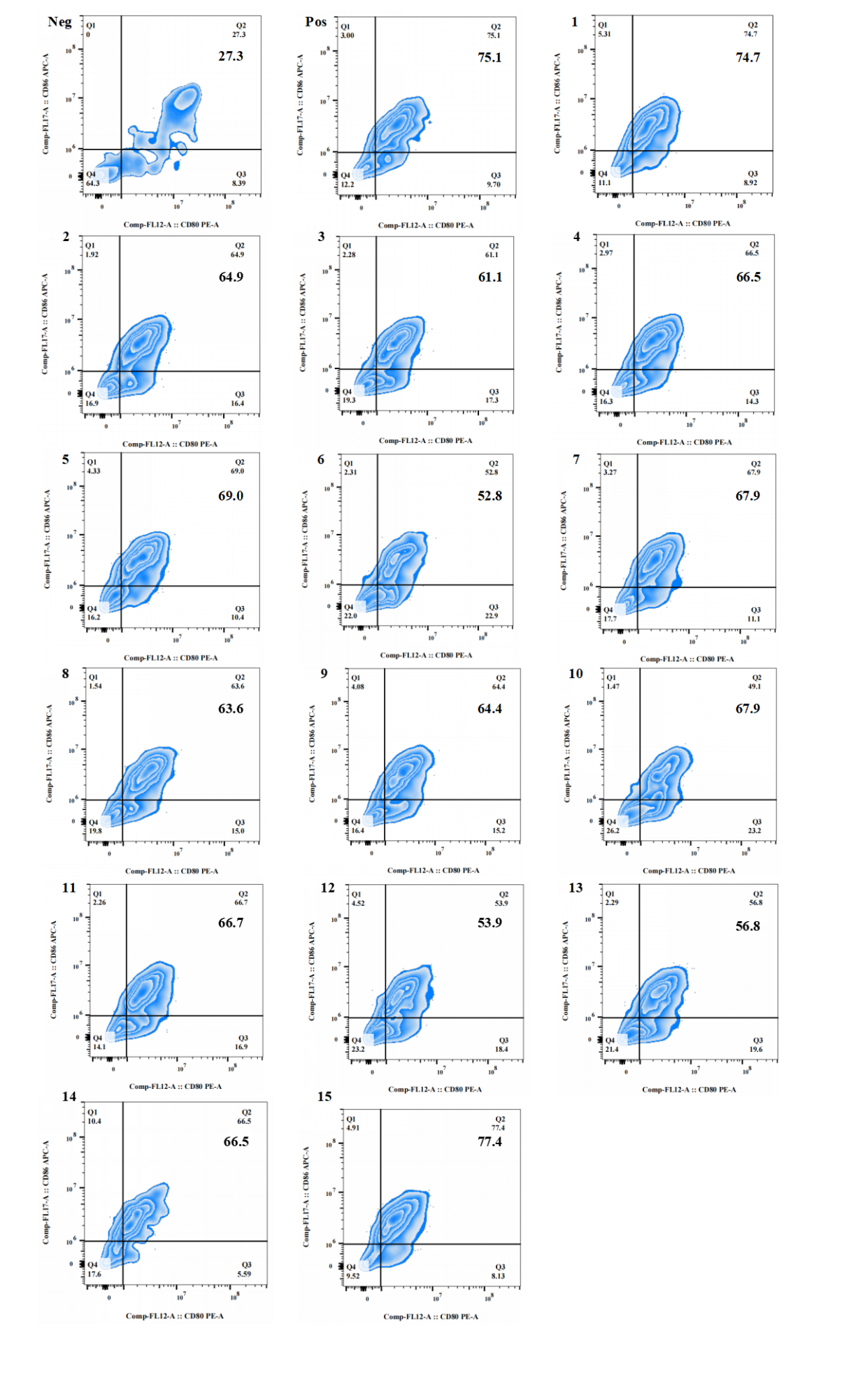


**Figure S10.** Flow cytometry representative images of the proportion of mature BMDCs stimulated by different candidate peptides, lipopolysaccharide, PBS.

**
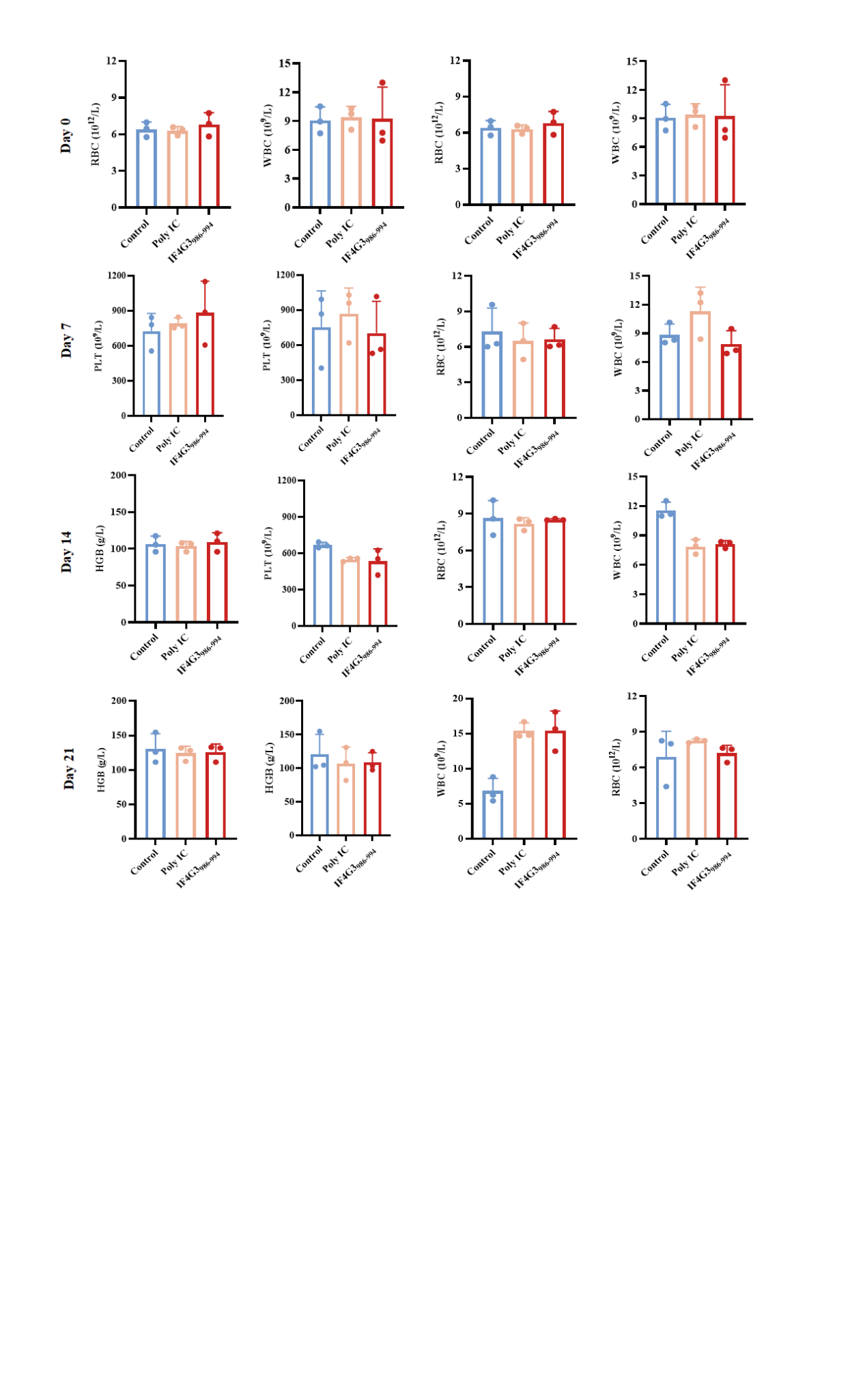
**

**Figure S11.** The levels of red blood cells, white blood cells, platelets, and hemoglobin in mice on days 0, 7, 14, and 21 after treatment with PBS/poly IC/poly IC + IF4G3_986-994_ (n =3, mean ± s.d.).

**
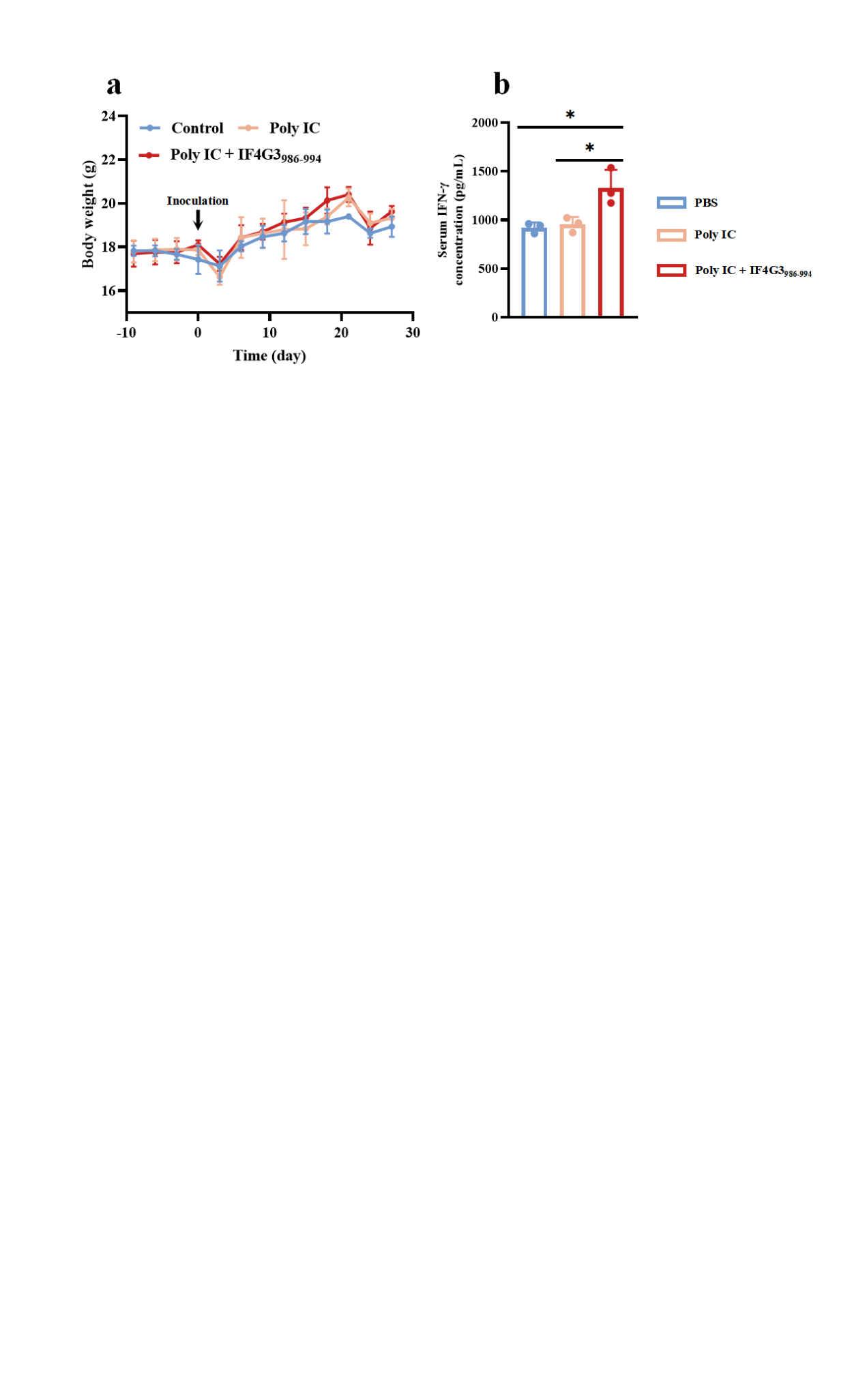
Figure S12.** (**a)** Body weights of mice during the observation period in IF4G3_986-994_ *in vivo* tumor vaccine experiments (n=4, mean ± s.d.). (**b)** Quantitative analysis of the concentration of IFN-γ in serum (n=3, mean ± s.d.).

1. **Supplementary Table**


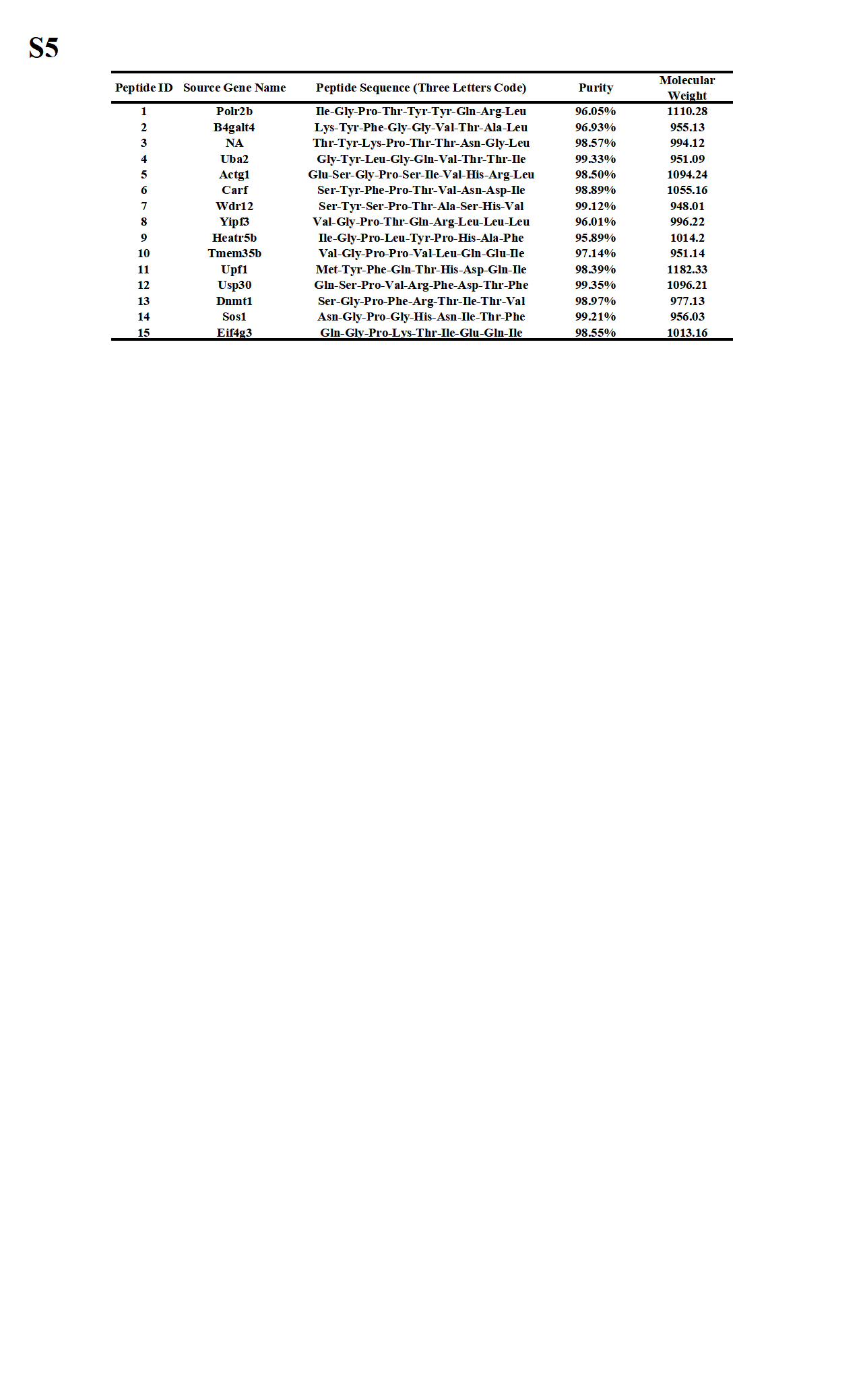


**Table S3.** The source gene name, peptide sequence (in 3-letter code), purity and molecular weight of candidate peptides.
